# A Simple Technique for Studying the Interaction of Polypropylene-Based Microplastics with Adherent Mammalian Cells Using a Holder

**DOI:** 10.3390/molecules30030516

**Published:** 2025-01-23

**Authors:** Magdalena Obłoza, Magdalena Ścibor, Marta Kaczor-Kamińska, Kamil Kamiński

**Affiliations:** 1 Faculty of Chemistry, Jagiellonian University, Gronostajowa 2 St., 30-387 Krakow, Poland; m.obloza@uj.edu.pl (M.O.); magdaslusarczyk98@gmail.com (M.Ś.); 2 Chair of Medical Biochemistry, Faculty of Medicine, Jagiellonian University Medical College, Kopernika 7 St., 31-034 Krakow, Poland; marta.b.kaczor@uj.edu.pl

**Keywords:** microplastics, cell cultures, 3D printing, colorectal adenocarcinoma

## Abstract

Microplastics pose a great challenge to human health and could prove to be the most dangerous environmental contaminant of the 21st century. The study presented here is an attempt at proposing a new methodology for studying the interaction of microplastics with adherent mammalian cells using aides. The disposable holders proposed here provide direct contact between microplastics (with a density lower than that of water) and cells in the course of culturing, which is necessary as we postulate the existence of an interaction. Using several microscopic methods (confocal fluorescence microscopy and scanning electron microscopy (SEM)), we have observed that this interaction causes a non-destructive penetration of the cell monolayer and adhesion of microplastics to the cell surface. The Caco-2 cells were used for the experiments. The said cells are the approximation of the digestive system, which, due to the presence of plastics in drinking water, is particularly vulnerable to direct interactions with these contaminants. Model microplastics were obtained by grinding pellets of chemically pure polypropylene. The imaging of cells in both space and on the surface was supplemented by an assay to determine the cell welfare in the studied microplastic-exposed models, which did not show the occurrence of apoptosis or necrosis after a 24 h exposure.

## 1. Introduction

Undeniably, polymers and the plastics derived from them are a group of compounds that have had the most spectacular impact on human life and its quality in the 20th century [1]. Today, we find synthetic polymers everywhere in our environment, from furniture and clothing to drug and cosmetic ingredients. This omnipresence of plastics must have also caused a dramatic increase in their presence in the ecosystem, whether due to unreasonable waste management or to other unintended processes [2,3]. Polymers of natural origin such as cellulose make up a significant share of the biomass present on Earth and have been used by humans since the birth of civilization, but their circulation in the environment is regulated via their biodegradability [4]. Synthetic polymers are mostly non-biodegradable; they accumulate in the ecosystem, which is a big problem [5]. An additional undesirable process that plastics undergo, regardless of their size, is the process of mechanical fragmentation, which leads to the formation of microplastics [6]. The latter, thanks to their small size and density, which is usually lower than that of water, easily penetrate seawater and groundwater and are difficult to remove from drinking water [7]. In the middle of the second decade of the 21st century, microplastics can be found everywhere, from the aforementioned water bodies to the air we breathe and rock sediments, hence the need to develop reliable methods to study their effects on living organisms [8].

Microplastics, as a new problem, are controversial due to the unverified detection and quantification methods [9] and being a strong argument for the potential transformation from an economy based on non-renewable sources to a sustainable one [10]. Problems with detecting microplastics in water samples result from the small number of these particles per unit volume, which does not mean that their quantity is small if we define it as a fraction of the mass or volume of the entire sample. This fact means that the detection of microplastics often requires a different approach and tactics than in the case of low-molecular-weight compounds [11]. However, according to today’s state of knowledge, there is no doubt that microplastics are present in water and pose a particular threat to aquatic organisms [12]. There are also confirmed literature reports of their presence in soil [13] and air [14]. These facts mean that the problem of microplastics does not only concern small aquatic organisms but, through their wide presence in the environment and interactions between organisms, broadly all organisms inhabiting our planet [15]. All these facts mean that today, in scientific circles, there is a broad consensus [16] that it is necessary to conduct research on the impact of microplastics on living organisms and the entire ecosystem, and the work presented here is a proposal for a new research model that will contribute to this.

Particularly important here is the effect on mammalian cells, and probably the most optimal biological model would be the use of cell lines [17]. There have been attempts to create such research models, but descriptions in the literature present contradictory results and the proposed models are characterized by great complexity and a high cost [18]. Therefore, the goal of this study was to propose a system that is relatively affordable, simple, and based on solutions that provide high repeatability (3D printing) and effectiveness for studying the effects of microplastics with densities lower than that of water on adherent mammalian cell lines.

The adherent cell line culture techniques commonly used today are not a perfect approximation of the conditions under which the mammalian cells grow [19]. The growth of cell monolayers on the bottom of multiwall plates or slides reflects flat (micro- and macro-scale) biological structures and does not provide knowledge of the growth conditions in more complex geometries. An example of such systems may be the coiled sections of the digestive system. Microplastics with a density lower than that of water will accumulate on the surface of the cell medium, which in practice in classic cultures (on the bottom of the culture vessel) prevents their contact with the surface of the cell monolayer. Such contact, however, can occur under physiological conditions in biological systems with a more complex geometry just like the previously mentioned digestive system. Microplastics present in the digestive tract contents can directly touch its walls in the intestinal tract’s corners [20].

There is no doubt that the negative impact of microplastics on cells can also result from other phenomena that do not require the direct contact of these particles with the cell surface. Here, we refer mainly to the release of partially water-soluble components from plastics with a proven toxicity, such as bisphenols [21] or other plastic fillers. This, however, is not the focus of this work and, due to the occurrence of such substances in microplastic-free drinking water for the last three decades, this topic is extensively covered in other research works. Therefore, in this study, we chose to use as a model the system of microplastics obtained by grinding (using a rotary wire brush) pellets of chemically pure polypropylene to minimize the number of variables. We also chose this polyolefin because it is widely used, there are no significant reports of its biodegradation occurring naturally, and the impact on cells when in the form of microplastics has been previously described in the literature [22].

**Figure 1:**
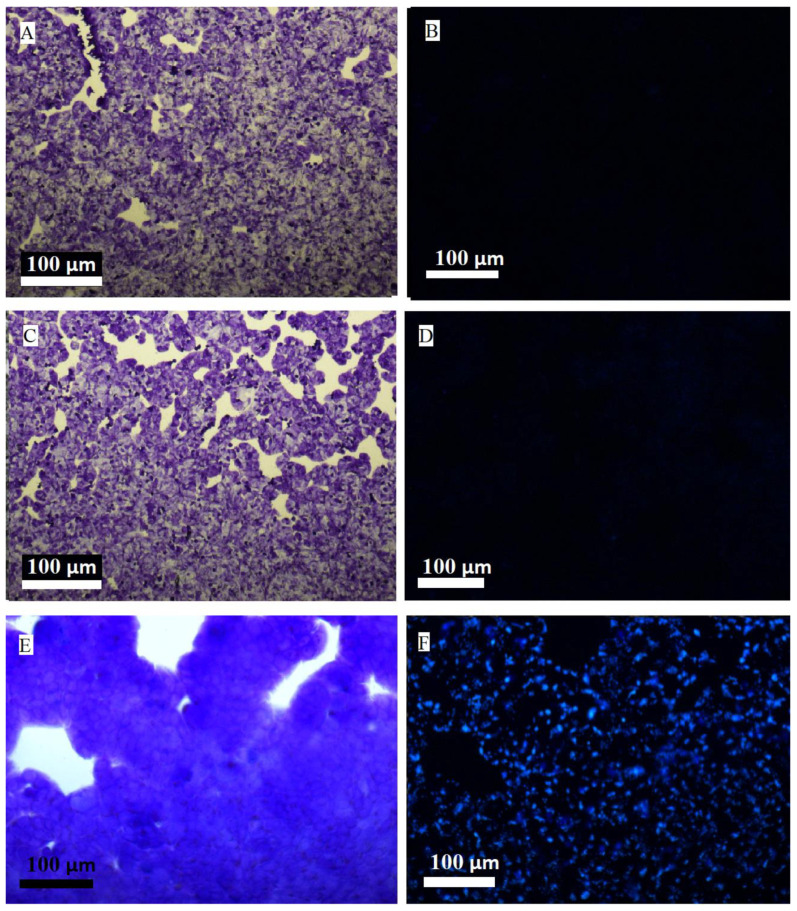
Visualizations of the Caco-2 cells on a slide additionally stained with crystal violet. On the left, transmitted light; on the right, simplified fluorescence microscopy (excitation with a mercury lamp). Sequentially from the top: (**A**,**B**) control without microplastics, (**C**,**D**) microplastics in the classic cell culture and microplastics in the cell culture with a holder, (**E**,**F**) microplastics labeled with Nile Red. Exposure time = 24 h. 5× magnification lens.

The basic premise presented here is a unique approach to modifying the geometry of the cell culture cultivation method (Figure 1) and, we believe, it is necessary to convincingly study the effects of microplastics on many biological systems. Achieving this goal in a reproducible and systematic manner is possible with the use of 3D printing [23] and biodegradable polylactic acid (PLA) [24] as a printing material. Three-dimensional printing ensures the reproducibility of the resulting shapes of the dedicated holder on which the glass slide with growing cells is mounted and the ability to scale the dimensions of this system. In addition, the most widely used and cheapest type of 3D printing was chosen by the present authors, namely Fused Deposition Modeling (FDM)/Fused Filament Fabrication (FFF) [25], as well as the most commonly used material, i.e., 1.75 mm PLA filament. This open approach to the methodology offers greater accessibility to the proposed methodology, and the biodegradability of PLA is an important aspect here because the study of microplastics cannot be an excuse to generate additional amounts of these pollutants.

Verification of the cell growth procedure and its interaction with microplastics was carried out using several microscopic methods (classical and confocal fluorescence microscopy and scanning electron microscopy (SEM)). Particular attention was paid to the fluorescence methods, which, after appropriate staining of microplastics (staining with Nile Red in hexane) [26], allow for determining the location of microplastics in three-dimensional space and, on this basis, for inferring their potential penetration into cells and the intercellular matrix.

These methods were complemented by SEM, which, without additional staining, allowed for confirming the presence of microplastics on the surface of the cell monolayer. In addition, it was possible to put the results obtained into a biological context by assessing the wellbeing of the cells by a live/dead (apoptosis/necrosis) fluorescence assay, which confirmed the absence of direct cytotoxicity. 

## 2. Results

### 2.1. Simplified Imaging with Classical Fluorescence Microscopy

The starting point for all the studies described here was confirming the fact that microplastics bind to the cell monolayer (Figure 1). Microplastics can be seen as bright dots in the fluorescence image (coinciding with the outline of the space in which the cells are present) using simplified fluorescence microscopy. The selection of the complexity, and thus cost, of the methods proposed in this paper was intended to provide greater access to the procedure presented here for imaging the interaction of microplastics with adherent cells.

The obtained images show that the incorporation of microplastics (visible as a multitude of blue dots with an outline that coincides with the cell outline in Figure 1E,F) into the monolayer only occurs using the cell culture supported by the 3D-printed PLA holder we propose in this publication. In the case of a system without plastics, no analogous objects are visible in the fluorescence image (Figure 1A,B), whereas, in the case of the classic cell culture, only minimal traces of blue spots are visible at the detection limit (Figure 1C,D). The detector settings for all measurements were identical. Unfortunately, this method does not allow for indicating how deeply the microplastics penetrate the cell layer and whether they enter the cytoplasm of the cells or are only confined to the intercellular matrix. In order to determine the localization of microplastics, it is necessary to use confocal microscopy, which we describe in the following sections.

### 2.2. Imaging Microplastics in the Three-Dimensional Space of a Cell Monolayer Using Fluorescence Confocal Microscopy

**Figure 2:**
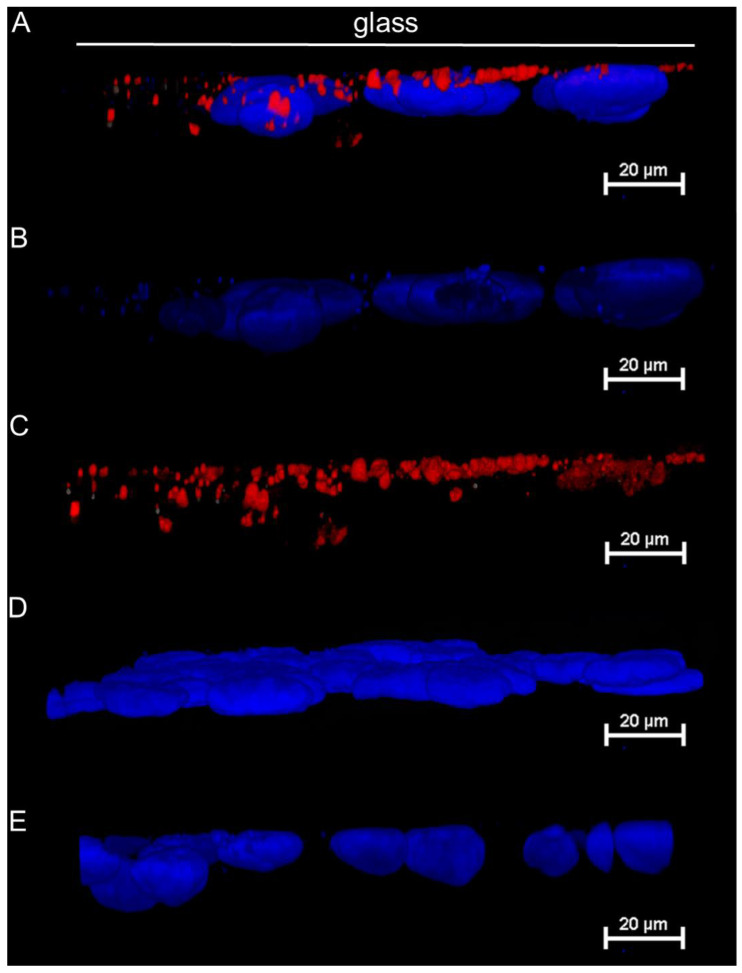
Confocal 3D images of the Caco-2 cells after exposure to microplastics for 24 h. The nuclei are stained blue, the microplastics are stained red. Cells incubated with microplastics for 24 h (cell culture with the holder): (**A**) merge channel, (**B**) blue channel, (**C**) red channel. Cells incubated with microplastics for 24 h (the classic cell culture): (**D**) merge channel. Culture of the Caco-2 cells with a holder and in the absence of microplastics: (**E**) merge channel.

To assess whether microplastics entered the cells and, if so, where they were located, we collected three-dimensional confocal images with different staining methods. In the first experiment, after incubation of the Caco-2 cells with fluorescent microplastics for 24 h in a dedicated holder, the cells were fixed with formaldehyde and the nuclei were stained with DAPI. The results show that microplastics were mainly localized in one plane, close to the surface of the glass on which the cells grew (Figure 2A,C). A precise determination on whether microplastics undergo cellular uptake and, if so, what their fate is in cells from the perspective of their potential localization in organelles is a complex problem requiring extensive research and is beyond the scope of this publication. The authors plan to do so in a subsequent publication. The nuclei remained unchanged, which, combined with the results of Section 2.3 showing no toxic effects on the cells in the presence of microplastics, may suggest that the plastics do not penetrate destructively into the cells.

The obtained results may suggest that microplastics derived from polypropylene, as chemically inert objects for cells, are pushed by the buoyancy force between each other and consequently penetrate the surface of the glass, which forms a physical boundary for them. This demonstrates that microplastics are not directly harmful to the cells lining the gastrointestinal tract, but such cells do not provide a barrier to microplastics and they can potentially penetrate the body and bloodstream. The long-term presence of microplastics combined with their accumulation may lead to undesirable effects, but this requires further research, which is beyond the scope of this paper. In the case of a classic culture without a dedicated prop holder, the stained microplastics are not visible (Figure 2D) in the image of the cell monolayer as in the case of the control system cultured in the absence of plastics (Figure 2E). This is further confirmation that the holder and the culture method described here are necessary for the interaction between microplastics and cells to occur.

### 2.3. Cytotoxicity of Microplastics—Investigating the Type of Cell Death

To assess whether microplastics are toxic and to determine the mechanism of cell death, we used an apoptosis detection kit. The method employed in this kit involves the use of two labels: Anexin-Cy3.18 (AnnCy3) binding to phosphatidylserine in the outer leaflet of the plasma membrane that initiates apoptosis, which is observed as red fluorescence, and the previously mentioned 6-Carboxyfluorescein diacetate (6-CFDA). There are three possible outcomes: (1) viable cells will stain only with 6-CF (green); (2) necrotic cells will stain only with AnnCy3 (red); and (3) cells initiating apoptosis will stain with both AnnCy3 (red) and 6-CF (green). We incubated the Caco-2 cells with microplastics for 24 h. This experiment showed no difference between the studied groups and no cytotoxicity was observed after the tested time period (Figure S1 in the Supplementary Materials).

### 2.4. Electron Microscopy (SEM) Images of Cells Cultured in the Presence of Microplastics

**Figure 3:**
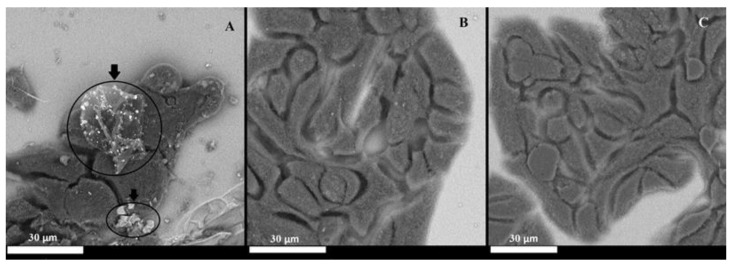
SEM images of the Caco-2 cells cultured after 24 h of exposure: (**A**) microplastics in geometry with the holder, (**B**) cell culture with the holder without microplastics, (**C**) classic cell culture with microplastics on the surface of the culture medium. Arrows indicate microplastics present on the microscopic image.

As the images below show, it is only when cells are cultured using dedicated systems that provide direct contact between the cells and microplastics that adsorption of plastics onto the surface of the cell monolayer occurs (Figure 3A). Microplastics can be seen as brighter polygonal objects with sharp edges (indicated by black arrows on Figure 3A) significantly different from rounded cells. Their size varies from more than ten to a few micrometers (Figure 3A). A large object can be seen in the center of the image, while a smaller object is visible near the lower edge of the image in the center part. This confirms the conclusions reached based on fluorescence microscopy imaging. In addition, the presence of microplastics on the surface when the sample was washed repeatedly with different solvents during fixation and preparation and subjected to a vacuum during the measurement itself proves that microplastics are strongly associated with the cell surface.

## 3. Discussion

The most important observation presented in the paper and not previously described in the literature is that the non-destructive penetration of the cell layer and adhesion of microplastics to the cell surface were only observed when dedicated conditions assisted by the 3D-printed PLA holder proposed in this work were used, whereas this was not the case under conditions where microplastics were only added to the medium and the cell culture was carried out in the classic manner. There are studies on the impact of microplastics in biological models but they present a broader picture and are based on environmental samples [27,28] and lack simplified models to study the mechanisms. The proposed cell culture method simulates the conditions found in the gastrointestinal tract and other tissues, where the physical properties of microplastics generate conditions where they are pressed into the tissue surfaces (for example, buoyancy due to their density being lower than that of water). This shows that microplastics derived from polypropylene, one of the most commonly used plastics, can penetrate the organism through the digestive system without directly damaging it. The lack of significant toxicity for microplastics obtained from this polymer for mammalian cells was also observed in other publications [22], which is consistent with our observation.

These findings may represent the beginning of research that will complement other important negative aspects resulting from the presence of microplastics in the gastrointestinal tract, such as changes in bacterial flora [29]. This is even more important as, due to the presence of microplastics in water, including drinking water, the digestive system may prove to be the main gateway to the human organism for these contaminants.

In this work, we have also shown that the presence of microplastics in the cell layer can be successfully observed using a variety of microscopic methods with varying degrees of complexity. For fluorescently stained microplastics, classic fluorescence microscopy with an excitation source in the form of a mercury lamp is sufficient to prove the presence of plastics, whereas their precise 3D localization requires confocal microscopy. Non-light microscopes, such as electron microscopes, can also detect the presence of microplastics on the surface of the cells.

## 4. Materials and Methods

### 4.1. Materials

Hexane (≥98% (GC), suitable for HPLC), Nile Red (for microscopy), ethanol (99% HPLC), polypropylene (granule, 4 mm nominal granule size, condition isotactic), hexamethyldisilazane (for GC derivatization, LiChropur, ≥99.0), glutaraldehyde (Grade I, 25% in H_2_O, specially purified for use as an electron microscopy fixative), phosphate-buffered saline (PBS, tablet, pH 7.2–7.6 (1 tablet/200 mL)), crystal violet (dye content ≥90%, certified by the Biological Stain Commission, powder), formaldehyde (for molecular biology, 36.5–38% in H_2_O), DAPI ready-made solution (1mg/mL for nuclear counterstaining in immunofluorescence microscopy), and the Annexin V-Cy3TM Apoptosis Detection Kit were purchased from Sigma-Aldrich (Poznan, Poland). For the isolation of microplastics, the isophore TM polycarbonate membrane (PC) (pore size, 1.2 μm; total diameter, 47 mm) was used (Millipore, Burlington, MA, USA). The human colorectal adenocarcinoma cell line (Caco-2) was purchased from the American Type Culture Collection (ATCC: HTB-37).

### 4.2. Obtaining of Microplastics

Polypropylene pellets weighing approximately 10 mg were ground using a wire brush disc (photo in Figure S2 in the Supplementary Materials) and an electric drill (PARKSIDE Cordless Rotary Tool PFBS 12 B3 (Parkside/Lidl Stiftung & Co. KG, Neckarsulm, Germany)) to simulate the processes that occur during plastic processing in an industrial environment. The grinding was carried out in a 250 mL tall beaker and then its walls were rinsed with 25 mL of distilled water. The resulting suspension was transferred to a Falcon 50 mL tube and centrifuged (1000 rpm for 5 min) to remove potential contaminants from the wire brush. The obtained supernatant, containing microplastics with a density lower than that of water, was used for further experiments.

### 4.3. Fluorescent Labeling of Obtained Microplastics

Fluorescent labeling was carried out following the procedure described previously in the literature [26]. The microplastic suspension was transferred to a filtration set equipped with a PC membrane (pore size, 1.2 μm) and the water was filtered out. In the next step, the microplastics present on the surface of the membrane were rinsed with 20 mL of ethanol and immersed in 2 mL of Nile Red 5 mg/L solution in hexane for 2 min. After that, the liquid was filtered and the microplastics were washed twice with 4 mL of hexane. The material on the membrane was air dried (inside a sterile laminar chamber) and transferred to a sterile vessel.

### 4.4. Classic Cell Culture in the Presence of Microplastics

The Caco-2 cells were maintained in Dulbecco’s Modified Eagle Medium (DMEM, high glucose, Life Technologies, Carlsbad, CA, USA) supplemented with 10% heat-inactivated fetal bovine serum (FBS, Life Technologies), penicillin (100 U/mL), and streptomycin (100 μg/mL) at 37° C in an atmosphere containing 5% CO_2_. The cells were seeded onto classic coverslips and, after 48 h and at about 70% coverage, were used for the experiment. To the microplastics obtained, 8 mL of the medium was added (for fluorescence imaging experiments, these were additionally fluorescently labelled according to the procedure described in Section 4.3) and equally distributed (without pipetting) to 4 wells (a 6-well plate) containing the slides with cells. After 24 h, the slides were rinsed with the medium to remove unbound microplastics and imaging was performed.

### 4.5. 3D Printing of a Holder for the Alternative Geometry Cell Culture Procedure

The 3D design was made using the freely available FreeCAD 0.21 program, converted in UltiMaker Cura, and printed on an Artillery Hornet 3D printer (Nozzle diameter: 0.4 mm). PLA Eco 1.75 mm Transparent from PlastSpaw (Rusinowice, Poland) was used as the filament. A ready-to-use *.stl file adapted to the parameters described here (a 6-well plate) is available for downloading as a part of the Supplementary Materials.

### 4.6. Cell Culture Procedure in an Alternative-Holder-Assisted Geometry

**Figure 4:**
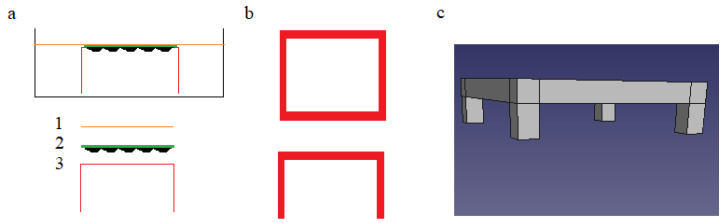
(**a**) Layout of the cell culture system with alternative geometry: 1—surface (liquid–gas boundary) of the cell culture medium, 2—a slide overgrown with cells, 3—a holder to ensure the correct position of the slide. (**b**) The slide holder used in the experiment, side and top view. (**c**) 3D presentation of the holder.

The coverslips were populated with cells in the same way as in Section 4.4. A suspension of microplastics in the medium was also prepared in a similar way and transferred to 4 empty wells. The PLA holders were then placed in the vessels in such a way that the top of the holder with the opening was at the same level as the liquid surface. The slide with the cells was then placed in the dish so that the cells were facing the surface of the medium (4.5 mL) and on the holder (see Figure 4 for an illustration of the shape of the holder and the way the coverslip was placed). After 24 h, the slides were rinsed with the medium to remove unbound microplastics and imaging was performed. An analogous experiment was carried out, but without microplastics in the medium, to obtain samples to illustrate the control experiments.

### 4.7. Fluorescence Microscopic Imaging

The glass coverslips with 70% of the surface covered with the cells (prepared as described in Section 4.4) were placed in 6-well plates containing the medium and fluorescently labeled microplastics for 24 h upside down on a holder, then washed three times with PBS and fixed using 4% formaldehyde. The cells were then washed three times with PBS and the nuclei (DNA) were stained with DAPI (0.1 μg/mL in PBS) for 20 min at room temperature. The cells were again washed with PBS and the coverslips with the cells were mounted on glass slides and sealed for confocal imaging. Fluorescent images were acquired using an A1-Si Nikon (Nikon, Tokyo, Japan) confocal laser scanning system coupled to a Nikon Ti-E inverted microscope (CLSM) using a Plan Apo 100×/1.4 Oil DIC objective. Three diode lasers (405, 488, and 561 nm) were used for excitation. Images were processed using the NIS-Elements AR 3.2 software (Nikon Europe BV, Amsterdam, The Netherlands) and ImageJ version 1.54m Fiji software (Madison, WI, USA) [30].

For imaging with a simplified fluorescence microscope (Nikon eclipse LV 100 optical microscope, Nikon, Tokyo, Japan), the processing cycle was limited only to the fixation procedure described above and additionally to staining with crystal violet [31] to improve the contrast for transmitted light microscopy using the following procedure.

### 4.8. Apoptosis Kit

Phosphatidylserine [PS] expression on the outside of apoptotic cells was shown using the Annexin V-Cy3TM Apoptosis Detection Kit (Sigma-Aldrich). Apoptotic cells can be distinguished from necrotic cells in various ways. The method employed in this kit involves the use of two labels. Anexin-Cy3.18 (AnnCy3) binds to phosphatidylserine in the outer leaflet of the plasma membrane, initiating the process of apoptosis, which is observed as red fluorescence. 6-Carboxyfluorescein diacetate (6-CFDA) is used to measure cell viability. When this non-fluorescent compound enters living cells, the esterases present therein hydrolyze it, producing the fluorescent compound 6-carbosyfluorescein (6-CF). This appears as a green fluorescence. There are three possible outcomes: (1) viable cells will stain only with 6-CF (green); (2) necrotic cells will stain only with AnnCy3 (red); and (3) cells initiating apoptosis will stain for both AnnCy3 (red) and 6-CF (green).

The Caco-2 cells on a coverslip (prepared analogously as in Section 2.4) were incubated with microplastics (prepared as described in Section 2.3 omitting the treatment of microplastics with the Nile Red solution) for 24 h at 37 °C upside down on a dedicated 3D-printed holder. The cells were then washed twice with 1 mL of PBS and three times with 50 µL of binding buffer (1 mM HEPES, pH 7.5, 14 mM NaCl, and 0.25 mM CaCl_2_). Then, 50 µL of the Double Label staining solution (1 µg/mL AnnCy3 and 500 µM 6-CFTA in binding buffer) was added and the cells were incubated for 10 min at room temperature. After staining, the cells were washed five times with 50 µL of binding buffer and coverslips were mounted on glass slides and sealed for confocal imaging. Fluorescence images were obtained using the A1-Si Nikon (Nikon, Tokyo, Japan) confocal laser scanning system coupled with the Nikon Ti-E inverted microscope and processed using the NIS-Elements AR 3.2 software (Nikon Europe BV, Amsterdam, The Netherlands).

### 4.9. SEM Measurements

The SEM images were recorded using a Phenom-World PRO environmental scanning electron microscope (Pik Instruments, Piaseczno, Poland). Samples were prepared by seeding the Caco-2 cells onto a glass slide and, after 24 h, transferring them to an inverted culture system on a holder as described in Section 4.6 with non-fluorescently labeled plastic (prepared as described in Section 4.3 excluding the treatment of microplastics with the Nile Red solution). The controls for these experiments were the systems cultured without microplastics and with microplastics but with a classic cell growth geometry; that is, a slide with cells resting on the bottom of the well. After 24 h of culturing the cells under these conditions, the slides were removed from the culture medium, washed with PBS solution at 37 °C, and fixed in a mixture of glutaraldehyde and PBS (1:7; *v*/*v*). Further processing of the sample to dehydrate it and fix its three-dimensional structure was carried out based on the procedure described in our previous work [31]. First, the sample was transferred to a 70% ethanol solution for a further 10 min. This procedure was repeated for successively increasing concentrations (80, 90, and 100%) of ethanol in water. The final step was to transfer the slide to hexamethyldisilazane (HMDS) for 5 min and air dry it for 10 min before final measurements.

## 5. Conclusions

The presence of microplastics in the ecosystem is a relatively new phenomenon and its effects on living organisms have not yet been comprehensively studied. The novelty of publications concerning this topic comes from the lack of established procedures for investigating potentially negative phenomena associated with the presence of microplastics and classic biological and physicochemical methods need to be adapted and validated. The work presented here addresses this problem and proposes a new strategy and procedures for applying selected microscopic methods to study the interaction of microplastics with densities less than that of water and mammalian cells. The authors showed that a modification of the classical method of culturing cells on coverslips using a holder provides optimal conditions for direct contact between such plastics and the cell line culture. The observations made indicate that, for the cells selected as the model (Caco-2 cells) and polypropylene-based microplastics, there was no direct destructive effect, but the plastics penetrated into the cell monolayer, which, subsequently, may have undesirable effects. The study also compared the microscopic methods used (SEM, confocal microscopy, and simple fluorescence microscopy), showing that fluorescence-based microscopy is the best method for investigating the interaction of microplastics and biological material, while confocal microscopy is optimal for obtaining a spatial image of the system.

## Data Availability

The raw data supporting the conclusions of this article will be made available by the authors on request.

## Supplementary Materials

The following supporting information can be downloaded at: https://www.mdpi.com/article/10.3390/molecules30030516/s1, Figure S1: Confocal images (magnification 100×) of Caco-2 cells after exposure to microplastics for 24 h using the holder (left) and the microplastic-free control (right). Live cells will only stain with 6-CF (green), necrotic cells will only stain with AnnCy3 (red), and cells starting the apoptotic process will stain both with AnnCy3 (red) and 6-CF (green). Figure S2: Device used to produce microplastics (PARKSIDE PFBS 12 B3 Cordless Rotary Tool with a mounted wire brush).
