# Supplementary material for: A Simple Technique for Studying the Interaction of Polypropylene-Based Microplastics with Adherent Mammalian Cells Using a Holder
Source: Molecules. 2025 Jan 23;30(3):516. doi: 10.3390/molecules30030516 (PMC11819840; doi:10.3390/molecules30030516)
Supplement: Supplementary file 1 [file molecules-30-00516-s001.zip › molecules-3339869-supplementary.pdf]

Supplementary material

A simple technique to study the interaction of polypropylene based microplastics with adherent mammalian cells using holder

Magdalena Obłoza [1], Magdalena Ścibor [1], Marta Kaczor-Kamińska [2] and Kamil Kamiński [1,*]

1 Faculty of Chemistry, Jagiellonian University, Gronostajowa 2 St., 30-387 Krakow, Poland
2 Chair of Medical Biochemistry, Faculty of Medicine, Jagiellonian University Medical College, Kopernika 7 St., 31-034 Krakow, Poland
* Correspondence: kaminski@chemia.uj.edu.pl; Tel.: +48 660589819

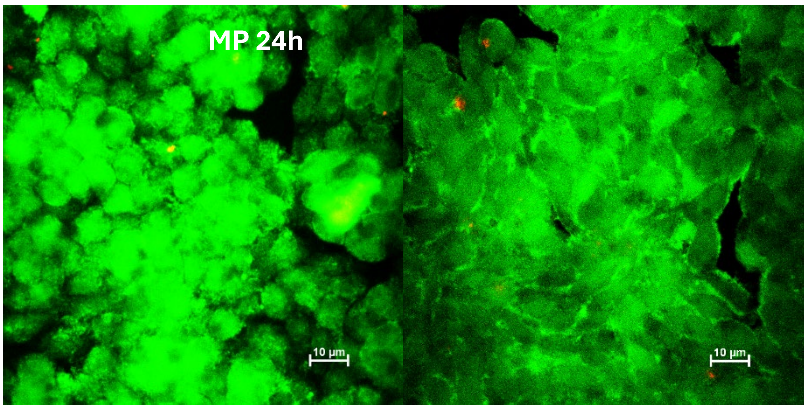

Figure S1. Confocal images (magnification 100x) of CaCO-2 cells after exposure to microplastics for 24h using the holder (left) and microplastic-free control (right). Live cells will only stain with 6-CF (green), necrotic cells will only stain with AnnCy3 (red), cells starting the apoptotic process will stain both with AnnCy3 (red) and 6-CF (green).

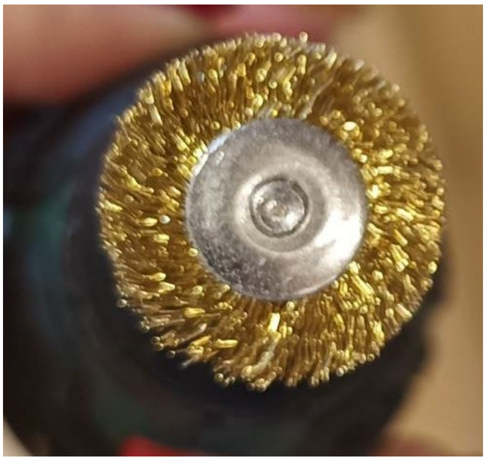

Figure S2. Device used to produce microplastics PARKSIDE Cordless Rotary Tool PFBS 12 B3 with mounted wire brush.
